# Identification of palliative care needs among people with dementia and its association with acute hospital care and community service use at the end-of-life: A retrospective cohort study using linked primary, community and secondary care data

**DOI:** 10.1177/02692163211019897

**Published:** 2021-05-31

**Authors:** Javiera Leniz, Irene J Higginson, Deokhee Yi, Zia Ul-Haq, Amanda Lucas, Katherine E Sleeman

**Affiliations:** 1 Cicely Saunders Institute of Palliative Care, Policy and Rehabilitation, King’s College London, London, UK; 2 Discover-Now, Imperial College Health Partners, London, UK

**Keywords:** Dementia, end-of-life, family practice, palliative care, primary health care, hospitalisation

## Abstract

**Background:**

Hospital admissions among people dying with dementia are common. It is not known whether identification of palliative care needs could help prevent unnecessary admissions.

**Aim:**

To examine the proportion of people with dementia identified as having palliative care needs in their last year of life, and the association between identification of needs and primary, community and hospital services in the last 90 days.

**Design:**

Retrospective cohort study using Discover, an administrative and clinical dataset from 365 primary care practices in London with deterministic individual-level data linkage to community and hospital records.

**Setting/participants:**

People diagnosed with dementia and registered with a general practitioner in North West London (UK) who died between 2016 and 2019. The primary outcome was multiple non-elective hospital admissions in the last 90 days of life. Secondary outcomes included contacts with primary and community care providers. We examined the association between identification of palliative care needs with outcomes.

**Results:**

Among 5804 decedents with dementia, 1953 (33.6%) were identified as having palliative care needs, including 1141 (19.7%) identified before the last 90 days of life. Identification of palliative care needs before the last 90 days was associated with a lower risk of multiple hospital admissions (Relative Risk 0.70, 95% CI 0.58–0.85) and more contacts with the primary care practice, community nurses and palliative care teams in the last 90 days.

**Conclusions:**

Further investigation of the mechanisms underlying the association between identification of palliative care needs and reduced hospital admissions could help reduce reliance on acute care for this population.

**What is already known about the topic?**

- People with dementia have a significant symptom burden and experience a rapid increase in non-elective admissions to hospital in their last months of life.
- People with dementia can experience barriers to accessing palliative care services, which may be exacerbated by difficulties in identification of people in their last year of life.
- Early recognition of palliative care needs is recommended to improve the quality of life for people approaching death, but the benefits of recognising palliative care needs for people with dementia are not known.

**What this paper adds**

- 1953 of 5804 (33.6%) people with dementia were identified as having palliative care needs in their last year of life; for 812 (14.0%) this occurred during the last 90 days of life, whereas 1141 people (19.7%) were identified as having palliative care needs prior to the last 90 days of life.
- Identification of palliative care needs in people with dementia is associated with more primary and community care contacts, including more community palliative care contacts, and fewer hospital admissions in the last 90 days of life.

**Implications for practice, theory or policy**

- Despite incentives to improve recognition of palliative care needs, the proportion of people with dementia identified as having palliative care needs remains low.
- Further research is needed to understand strategies to help primary care physicians to improve early recognition of palliative care needs in their patients with dementia.
- The early recognition of palliative care needs among people with dementia could be an important component of interventions aiming to reduce unnecessary unplanned admissions to hospital at the end of life.

## Introduction

Globally, the burden of serious health-related suffering associated with dementia is projected to increase four-fold in the next 40 years.^1^ In England and Wales the number of people dying with dementia is projected to increase by 270% by 2040, due to improvements in life expectancy and an increase in the number of people aged over 65.^2^

People with dementia can receive poor quality care at the end of life, including low levels of symptom assessment,^3,4^ in spite of experiencing similar levels of distressing symptoms as people with other chronic conditions.^5,6^ Adopting a palliative care approach can benefit people with dementia, but structures of community service provision and difficulties recognising palliative care needs due to multiple comorbidities, cognitive changes, communication difficulties and the pattern of slow incremental decline may be barriers to this.^7–11^ In addition, dementia has been traditionally under-recognised as a terminal illness.^9,12^ In people with cancer, Chronic Obstructive Pulmonary Disease and Chronic Heart Disease, recognition of palliative care needs is associated with better quality of life, contacts with primary care services, more chances to die at home and avoidance of overly aggressive treatment near the end of life including hospital admissions.^13–15^ The extent to which people with dementia are identified as having palliative care needs, and the benefits of this, are not clear.

Transitions from community to hospital settings for people with dementia near the end of life can be distressing for patients and careers,^16^ and are associated with increased risk of delirium, falls, cognitive and functional decline, readmissions and death.^12,17,18^ In spite of these negative outcomes, end-of-life transitions are frequent among people with dementia.^19^ Multiple transitions in the last 90 days of life have been suggested as an indicator of poor end-of-life care in this population.^19,20^

The aim of this study was (1) to explore the proportion and characteristics of people with a diagnosis of dementia who are identified as having palliative care needs during the last year of life, and (2) to examine the association between identification of palliative care needs before the last 90 days and multiple non-elective hospital admissions, primary and community care contacts during the last 90 days of life. We hypothesised that identification of palliative care needs would be associated with fewer hospital admissions close to death, and with a higher number of community palliative care team contacts based on results from previous studies.^13–15^

## Methods

### Design

This is a retrospective cohort study using the Discover dataset. The Discover dataset is a platform that enables researcher access to pseudonymised patient-level data drawn from the Whole Systems Integrated Care (WSIC) local data warehouse for research purposes. Discover dataset is maintained and interrogated on a secure server and extracts of data are aggregated in compliance with the Information Governance suppression rule where numbers below five are annotated as <5. In this process, the de-identified data is rendered anonymised by stripping out any information that would allow re-identification of an individual’s identity.

This dataset is one of Europe’s largest linked longitudinal costed de-identified datasets and includes over 2.6 million patients who live and are registered with a general practitioner in North West London. The database is spread across eight Clinical Commissioning Groups (CCGs) accounting for 95% of the total North West London population. This dataset is fed by data from over 400 provider organisations including 365 primary care practices, two mental health and two community trusts and all acute providers attended by North West London patients. A deterministic individual-level linkage is used between different data providers.^21^ The age and gender distribution and prevalence of long-term conditions of the Discover population are similar to the rest of London and the UK. However, a higher proportion of Asian, Black and mixed ethnicity than the UK population is observed.^21^

### Population

Adults (aged 18 or over) included in the Discover dataset with a diagnosis of dementia, who died between 1st April 2016 and 31st March 2019 were included. Decedents were identified using the register status codes for death status specified by the Discover dataset. Primary care practices in the UK use standard clinical codes called Read codes to record patients’ findings and procedures. More recently, primary care practices have been transitioning to a new clinical terminology called SNOMED CT to replace Read codes. During this transitioning period, practices in North West London have reported both types of codes to the WSIC.^22^ People with a diagnosis of dementia recorded in primary care records or hospital in-patient records were identified using Read codes and International Statistical Classification of Diseases and Related Health Problems (ICD) 10 codes respectively. Read codes were based on the Quality and Outcomes Framework (QoF) Rules for dementia v 37.0 2017/2018 (Supplemental Material).^23^ The date of death was derived from Read codes in primary care records and the discharge date for patients who died in hospital. People whose date of death was not found in hospital or primary care records were removed (Supplemental Material).

### Exposure of interest: Identification of palliative care needs before the last 90 days of life

The Quality and Outcomes Framework (QoF) is a voluntary pay-for-performance scheme introduced in 2004 in the UK for primary care practices. In 2006, the scheme incorporated a quality indicator that encouraged primary care practices to identify people with palliative care needs and to form a practice-based register of those patients. We used the first recorded Read code specified for the Palliative Care QoF register to determine identification of palliative care needs.

For our main analysis, we defined identification of palliative care need before the last 90 days of life to ensure our exposure variable occurred before the primary outcome was measured (0 if no Palliative Care QoF code was identified or if the date was in the last 90 days of life; 1 if the code was identified before the last 90 days of life).

### Primary outcome

The primary outcome was multiple non-elective admissions to hospital in the last 90 days of life (0 if no, 1 if yes), defined based on the work of Gozalo et al.^20^ as either more than two non-elective admissions for any reason or more than one non-elective admission for respiratory infection, urinary tract infection, dehydration or sepsis in the last 90 days of life. Non-elective admissions were identified through in-hospital records using start dates and admission method (see Supplemental Material).

### Secondary outcomes

Secondary outcomes included community and primary care contacts in the last 90 days of life.

- Community care: the number of contacts with community nurses, community palliative care teams, physiotherapists, speech and language therapists and occupational therapists was derived from unique episodes of contact based on the date of the contact and the corresponding description of the service. Contacts with speech and language therapists and occupational therapists were grouped with physiotherapist into rehabilitation teams due to low numbers. Duplicates based on the date of the contact, attendance and description of the service were removed and non-attendant contacts were excluded (Supplemental Material).
- Primary care practice: As the primary care practice record dataset does not directly report appointments or consultations, the number of contacts at the primary care practice was derived from Read codes from the primary care records using a similar approach reported by Kontopantelis et al.^24^ Contacts were classified as face-to-face or telephone consultations. It was not possible to identify whether the contact in the practice was with a doctor or another healthcare professional (Supplemental Material). In instances where people had two or more consultations within a day, it was assumed a single consultation took place, to reduce the likelihood of including duplicate records.^24^

### Covariates

- Demographics: Age at death, gender, ethnicity and Index of Multiple Deprivation were extracted from Discover dataset records for each individual. The 2015 Index of Multiple Deprivation, an area-level indicator of socioeconomic position, was derived at Lower Super Output Areas (LSOAs) level from the last registered address. We identified individuals who were defined by Discover primary care dataset as living in a care home based on the latest patient record. The year of death was classified based on administrative years (1st April to 31st March of the following year).
- Illness-related: the number of comorbidities was calculated using the count of chronic disease from Quality and Outcomes Framework rules obtained from Read codes in the primary care dataset.^23^ This approach to assessing multimorbidity performs similarly to other multimorbidity indexes such as the Charlson Index or the expanded Diagnosis Clusters count to predict 3-year mortality and consultations with primary care services,^25^ and has been used in other studies using primary care records in England.^26–28^

### Analysis

Descriptive statistics were used to describe the sociodemographic and clinical characteristics of the study population.

We used generalised estimating equations (GEE) to estimate the unadjusted and multivariate association between identification of palliative care needs before the last 90 days and multiple hospital admissions in the last 90 days of life. A Poisson family with log link function and exchangeable correlation structure was used to provide risk ratios (RR) and 95% confidence intervals. Robust variance estimates were used, with data clustered in primary care practices where people were registered. For the multivariate model, we adjusted by sociodemographic and clinical characteristics selected according to a priori hypotheses, and significance in unadjusted analysis (*p* ⩽ 0.05). We used a Poisson regression with robust error variance rather than odds ratios, as odds ratios do not approximate to risk ratios when the probability of the outcome is high, and may be misinterpreted.^29^

We used zero-inflated negative binomial regressions to estimate the unadjusted and multivariate association between identification of palliative care needs before the last 90 days and each of the secondary outcomes. We chose the zero-inflated negative binomial regression to account for the excess zeros and the overdispersion of the data. Data was clustered in primary care practices where people were registered and the incidence risk ratio (IRR) and 95% confidence intervals were reported. For the negative binomial models, we adjusted by age, gender, living in a care home and number of comorbidities. We used IMD quintiles and the other covariables in the negative binomial model to adjust the logit part of the model. We excluded ethnicity from the multivariate models to avoid bias in the sample due to the number of missing values. We performed a sensitivity analysis to investigate the impact of adding ethnicity in the models.

The analysis was performed using RStudio 3.6.0.

### Ethics statement

The source database is approved for secondary analysis by the West Midland-Solihull Research Ethics Committee (reference 18/WM/0323).

## Results

### Characteristics of the cohort

A total of 47552 people with a diagnosis of dementia recorded were identified. Of these, 5804 people had a date of death between 1st April 2016 and 31st March 2019 and were therefore included in the analysis. People between 86 and 95 years old comprised 50.7% of the sample. 3278 (56.5%) were women, 44.3% were of white ethnicity and 1306 (22.5%) people were reported as living in a care home before dying. Overall, 2576 (44.4%) people had three or more comorbidities, most commonly hypertension (64.5%) (Table 1).

**Table 1.** Population characteristics for those with and without identification of palliative care needs before the last 90 days of life.

	Total sample (*n* = 5804)	Identification of palliative care needs before last 90 days	
		No (*n* = 4663)	Yes (*n* = 1141)
Distribution of age (years)			
⩽75	379 (6.5%)	306 (6.6%)	73 (6.4%)
76–85	1479 (25.5%)	1241 (26.6%)	238 (20.9%)
86–95	2945 (50.7%)	2359 (50.6%)	586 (51.4%)
>95	1001 (17.3%)	757 (16.2%)	244 (21.4%)
Gender			
Female	3278 (56.5%)	2589 (55.5%)	689 (60.4%)
Male	2526 (43.5%)	2074 (44.5%)	452 (39.6%)
Ethnicity			
White	2573 (44.3%)	2049 (43.9%)	524 (45.9%)
Black	358 (6.2%)	295 (6.3%)	63 (5.5%)
Asian	864 (14.9%)	708 (15.2%)	156 (13.7%)
Mixed	922 (15.9%)	738 (15.8%)	184 (16.1%)
Not know	1087 (18.7%)	873 (18.7%)	214 (18.8%)
IMD quintiles			
1 (Most deprived)	1047 (18.0%)	828 (17.8%)	219 (19.2%)
2	1751 (30.2%)	1428 (30.6%)	323 (28.3%)
3	1537 (26.5%)	1251 (26.8%)	286 (25.1%)
4	978 (16.9%)	754 (16.2%)	224 (19.6%)
5 (Most affluent)	491 (8.5%)	402 (8.6%)	89 (7.8%)
Lived in a care home			
Yes	1306 (22.5%)	909 (19.5%)	397 (34.8%)
Year of death			
2016–17	1855 (32.0%)	1525 (32.7%)	330 (28.9%)
2017–18	2022 (34.8%)	1621 (34.8%)	401 (35.1%)
2018–19	1927 (33.2%)	1517 (32.5%)	410 (35.9%)
Number of QoF comorbidities			
0	1019 (17.6%)	842 (18.1%)	177 (15.5%)
1	936 (16.1%)	746 (16.0%)	190 (16.7%)
2	1273 (21.9%)	1042 (22.3%)	231 (20.2%)
⩾3	2576 (44.4%)	2033 (43.6%)	543 (47.6%)
Cancer (QoF)			
Yes	1091 (18.8%)	784 (16.8%)	307 (26.9%)
Diabetes (QoF)			
Yes	1687 (29.1%)	1380 (29.6%)	307 (26.9%)
Hypertension (QoF)			
Yes	3742 (64.5%)	3018 (64.7%)	724 (63.5%)
COPD (QoF)			
Yes	687 (11.8%)	531 (11.4%)	156 (13.7%)
Stroke (QoF)			
Yes	1100 (19.0%)	854 (18.3%)	246 (21.6%)
Coronary heart disease (QoF)			
Yes	1473 (25.4%)	1156 (24.8%)	317 (27.8%)

QoF: quality of life framework; COPD: chronic obstructive pulmonary disease; IMD: index of multiple deprivation.

### Identification of palliative care needs through the last year of life

**Figure 1.**
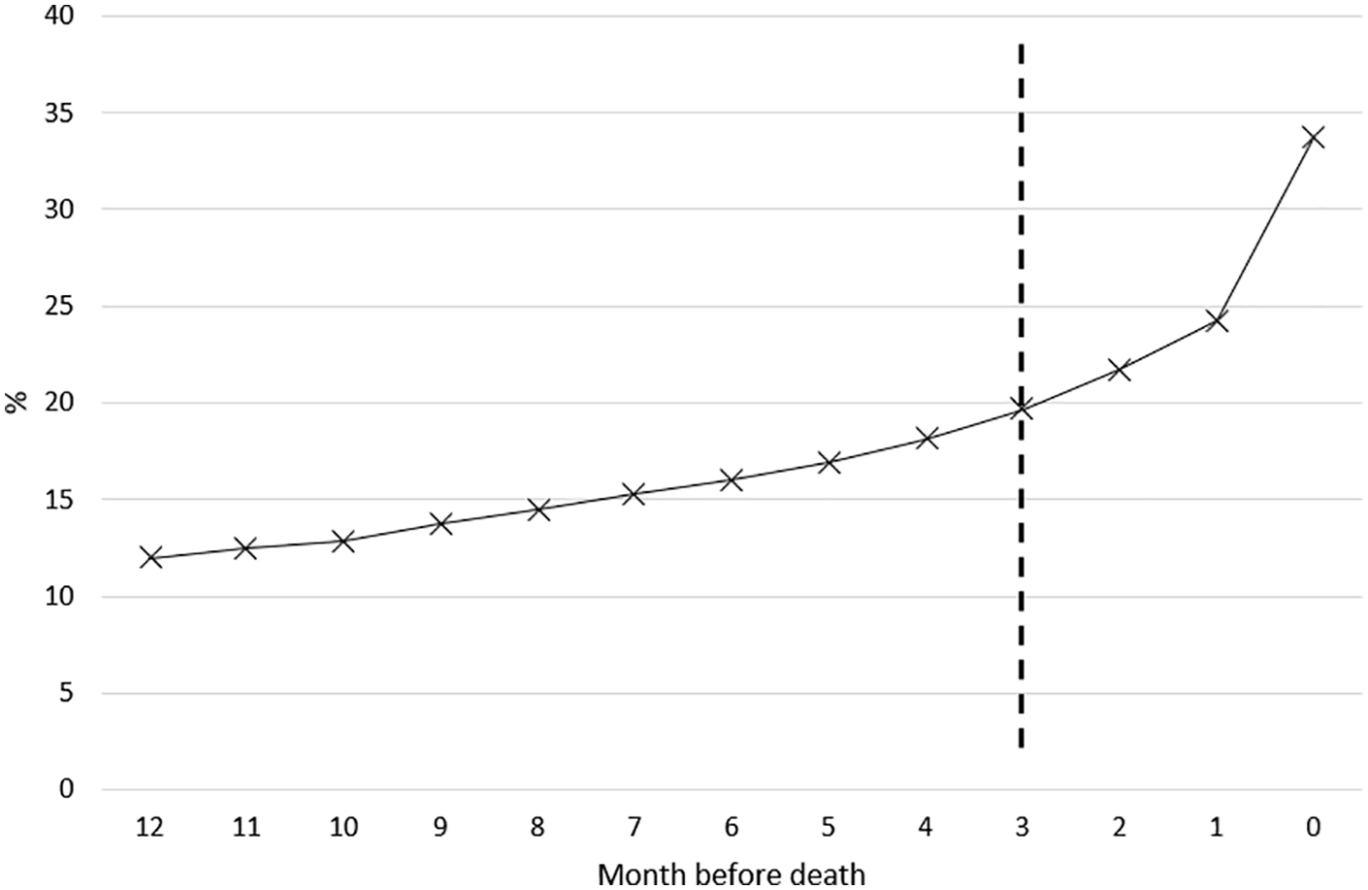
Cumulative proportion of people with dementia that have been identified with palliative care needs by month before death.

Overall, 1953 of 5804 (33.6%) people had a record of palliative care needs identification: for 812 (14.0%) this occurred during the last 90 days of life, whereas 1141 (19.7%) were identified prior to the last 90 days of life (Figure 1).

People with identification of palliative care needs before the last 90 days were more likely to be older, female, to live in a care home and to have more comorbidities (Table 1).

### Multiple non-elective admissions at the end of life

Overall, 737 (12.7%) people in the cohort experienced multiple non-elective hospital admissions in the last 90 days of life. After adjusting for confounders, people with identification of palliative care needs before the last 90 days were less likely to have multiple hospital admissions during the last 90 days of life (RR 0.70, 95% CI 0.58–0.85) (Table 2). In the sensitivity analysis, adding the ethnicity variable did not significantly change the results (Supplemental Material).

**Table 2.** Adjusted association between identification of palliative care needs before the last 90 days and primary and secondary outcomes.

Primary outcome	Total sample (*n* = 5804)			Exposure variable						Adjusted model*	
				Identification of palliative care needs before last 90 days							
				No (*n* = 4663)			Yes (*n* = 1141)				
	*n* (%)			*n* (%)			*n* (%)			RR	95% CI
Multiple non-elective hospital admissions in the last 90 days of life (Yes)	737 (12.7)			639 (13.7)			98 (8.6)			0.70	0.58–0.85
Secondary outcomes	Number people with 1 or more events *n* (%)	Number of events	Incidence rate per 100 people	Number people with 1 or more events *n* (%)	Number of events	Incidence rate per 100 people	Number people with 1 or more events *n* (%)	Number of events	Incidence rate per 100 people	IRR	95% CI
Face-to-face contacts with GP practice	1132 (19.5)	1837	32	865 (18.6)	1368	29	267 (23.4)	469	41	1.35	1.12–1.62
Telephone contacts with GP practice	2658 (45.8)	8756	151	2104 (45.1)	6855	147	554 (48.6)	1901	167	1.16	1.02–1.31
Contacts with community nurses	2782 (47.9)	32459	559	2181 (46.8)	24665	529	601 (52.7)	7794	683	1.34	1.18–1.53
Contacts with community palliative care teams	403 (6.9)	1178	20	257 (5.5)	671	14	146 (12.8)	507	44	2.06	1.49–2.86
Contacts with physiotherapy teams in the community	589 (10.1)	1623	28	449 (9.6)	1311	28	140 (12.3)	312	27	1.00	0.75–1.33
Contacts with rehabilitation teams in the community	638 (11.0)	1727	30	496 (10.6)	1406	30	142 (12.4)	321	28	0.97	0.74–1.27

RR: relative risk; IRR: incidence risk ratio.

The primary outcome section shows the results from the adjusted model using a generalised estimating equation with a Poisson family, log link function and robust variance to estimate the association between identification of palliative care needs before the last 90 days and multiple hospital admissions in the last 90 days of life (examined as a dichotomous outcome). The secondary outcome section shows the results from the adjusted models using a zero-inflated negative binomial regression to estimate the association between identification of palliative care needs before the last 90 days and number of contacts with primary care practice and community care services in the last 90 days of life (examined as a continuous outcome).

* Adjusted by age, gender, living in care home and number of comorbidities.

### Contacts with primary and community care providers at the end of life

Among the 5804 decedents with dementia, 1132 (19.5%) and 2658 (45.8%) had at least one face-to-face or telephone contact with the primary care practice in the last 90 days of life. 2782 (47.9%) people in the cohort had at least one contact with community nurses in the last 90 days. Four hundred and three (6.9%) people had at least one contact with community palliative care teams, 589 (10.1%) with physiotherapists and 638 (11.0%) with rehabilitation teams.

People with identification of palliative care needs before the last 90 days had a higher number of face-to-face, telephone, community nurses and palliative care team contacts in the last 90 days of life, after adjusting for confounders (Table 2). No significant difference in the number of contacts with physiotherapy and rehabilitation teams were found (Table 2).

## Discussion

### Main findings

In this sample of decedents with a diagnosis of dementia, one in three people was identified as having palliative care needs, and for one in five this identification occurred before the last 90 days of life. People with identification of palliative care needs before the last 90 days had more contacts with their primary care practice, community nurses and community palliative care teams, and were less likely to have multiple non-elective admissions to hospital in their last 90 days of life.

In our cohort, one third of people were recognised as having palliative care needs in the last year of life. A study in six primary care practices in Scotland published in 2012 showed that only 8% of people who died with dementia were identified as having palliative care needs in the last 6 months of life.^30^ It is possible that the introduction of the Palliative Care Quality and Outcomes Framework financial incentive in 2006, contributed to an increasing recognition of palliative care needs. Prospective studies show that people with dementia have a prevalence of restricting symptoms close to 50% in the last months of life^5,6^ and therefore, it is likely the number of people with dementia who would benefit from a palliative care approach remains under-recognised.

We found identification of palliative care needs before the last 90 days was associated with a lower risk of having multiple non-elective admissions to hospital during the last 90 days of life. Other studies in people who died of cancer, COPD or CHF found similar results.^15^ By recognising palliative care needs, proactive care planning such as advance care planning or assessment of symptoms may help improve the quality of community care and reduce unnecessary admissions to hospital. One potential mechanism highlighted in our study to explain this association is that identification of palliative care needs is associated with more community care and palliative care contacts.

Our study shows that people who are identified as having palliative care needs have more contacts with community palliative care teams and nurses in the community. These findings are similar to those of a study in Dutch patients, where recognition of dying before patients’ last week of life was associated with more primary care contacts during the last week of life.^13^ Primary care visits have been associated with fewer hospital days at the end of life, fewer preventable hospitalisations and higher odds of dying with home or hospice care.^31–33^ Identifying people who would benefit from a palliative care approach might improve their access to different healthcare providers, such as palliative care teams, which in turn could lead to a better quality of care.

Early identification of palliative care needs has been identified as a challenge by primary care practitioners. Family physicians report barriers such as not having knowledge, skills and experience relating to palliative care needs, lack of time and continuity of care, lack of collaboration with other caregivers and specialists, and fears of discussing end-of-life care issues with patients.^30,34,35^ Improving training for primary healthcare professionals could help to improve the recognition of palliative care needs for people with dementia, as well as improving continuity of care and coordination between primary and secondary care.

### Strengths and limitations

It was not possible to determine the appropriateness of hospital admissions for individual patients. While some hospital admissions at the end-of-life might be necessary, having multiple admissions has been proposed as an indicator of potentially over-aggressive care at the population level. We did not have information on the quality of the care provided by community and primary care service, symptom burden or preferences, which are likely to have an impact on unplanned hospital admissions.

As the Discover dataset is not linked with death registers, the date of death was derived from primary care and hospital records. Therefore, some level of inaccuracy in the date of death used might be expected. However, a good level of agreement between the date of death registered in primary care records and hospital records was found for people who died in hospital. Information on primary care contacts was derived from Read codes. While the approach used has been reported in previous studies,^24,36^ it is likely underestimating the number of consultations in primary care.

The use of clinical and administrative data presents opportunities and challenges. The quality of health care records relies on professionals’ and administrators’ skills and training, which varies across sectors and can be influenced by economic incentives.^37^ Changes in coding practices and financial incentives such as the introduction of the QoF in England, are likely to have had an impact on the number of people identified as having palliative care needs.^38^

This study has important strengths. This is one of the first studies to use linked data across primary, community and hospital services in England to explore end-of-life care. The Discover dataset holds comprehensive data for over 2 million people, providing an opportunity to explore healthcare use within the wider health system. Linked data from primary and community care services allows us to avoid the bias of information coming only from secondary care, and enables triangulation of information between different sources.

## Conclusion

In this cohort of people dying with dementia, identification of palliative care needs in the last year of life was associated with more contacts with primary and community care professionals, and a lower risk of multiple non-elective admissions to hospital in their last 90 days of life. Only 19.7% of people with dementia had their palliative care needs formally identified before their last 90 days of life. Further research is needed to understand strategies to help primary care physicians to improve recognition of palliative care needs in their patients with dementia and to understand the mechanisms that lead to a lower risk of multiple hospital admissions in this population.

## Supplemental Material

sj-pdf-1-pmj-10.1177_02692163211019897 – Supplemental material for Identification of palliative care needs among people with dementia and its association with acute hospital care and community service use at the end-of-life: A retrospective cohort study using linked primary, community and secondary care data Click here for additional data file. Supplemental material, sj-pdf-1-pmj-10.1177_02692163211019897 for Identification of palliative care needs among people with dementia and its association with acute hospital care and community service use at the end-of-life: A retrospective cohort study using linked primary, community and secondary care data by Javiera Leniz, Irene J Higginson, Deokhee Yi, Zia Ul-Haq, Amanda Lucas and Katherine E Sleeman in Palliative Medicine
