# Supplementary material for: Identification of palliative care needs among people with dementia and its association with acute hospital care and community service use at the end-of-life: A retrospective cohort study using linked primary, community and secondary care data
Source: Palliat Med. 2021 May 31;35(9):1691–700. doi: 10.1177/02692163211019897 (PMC8532216; doi:10.1177/02692163211019897)
Supplement: sj-pdf-1-pmj-10.1177_02692163211019897 – Supplemental material for Identification of palliative care needs among people with dementia and its association with acute hospital care and community service use at the end-of-life: A retrospective cohort study using linked primary, community and secondary  [file sj-pdf-1-pmj-10.1177_02692163211019897.pdf]

Supplementary material

Table 1. List of codes used to identify death of death and patients with dementia in primary care and hospital records.

	Read Codes v2	ICD10 codes
Date of death	22J.., 9491., 9495., 94G.., 8HG.., 9493., 94E.., 946.., 94Z.., ZV680, 94..., 949A., 949.., 9431., 9442., 9451., 9452., 9453., 946.., 9492., 9494., 9496., 9497., 9498., 9499., 949B., 949C., 949D., 949E., 949F., 949G., 949H., 949J., 949Z., 94D.., 94G..	
Diagnosis of dementia	F110 to F112, E02y1, E041., Eu041, F116.,<br>F118., F21y2, A410., Eu107, F11x7, Eu02%, E00%, Eu01%, E012%, Eu00%, A411%	F000 to F03x<br>G30 to G30.9<br>G31 to G31.8<br>G91.2
Multiple admission in last 90 days		>2 hospital admissions with a Epistart date >= (date of death – 90) +<br>>1 hospital admissions with a Epistart date >=(date of death – 90) & primary diagnosis of:<br>Urinary tract infection (ICD-10 code N39.0), Respiratory tract infection (ICD-10 codes J00-J22.9, J69.0), Sepsis (ICD-10 codes A40.0-A40.9, A41.0-A41.9, A02.1, A26.7, A22.7, A32.7, A42.7), and dehydration (ICD-10 code E86)

Table 2. List of codes used to derive community and primary care contacts

District nurse contacts	ServiceReferenceCostDescription= 'District Nurse' or 'District Nurse, Adult, Face to face' or 'District Nursing services: Adult' OR<br>Service Reporting Line = 'District Nursing' or 'District Nursing (H&F, K&C, W)' or 'District Nursing – AWC'
Palliative care community team contacts	Service Reporting line = 'Palliative Care' or 'Palliative Care Service' OR<br>ServiceReferenceCostDescription= 'Palliative / Respite Care: Adult' or 'Specialist Nursing, Palliative/Respite care, Adult, Face to face'
All nurses	DISTRICT NURSE CONTACTS +<br>ServiceReferenceCostDescription= 'Cardiac Nursing / Liaison: Adult' or<br>'Other Specialist Nursing' or<br>'Other Specialist Nursing, Adult, Face to face' or<br>'Specialist Nursing – Asthma and Respiratory Nursing/Liaison' or<br>'Specialist Nursing – Active Case Management (Community Matrons)' or<br>'Specialist Nursing – Cardiac Nursing/Liaison, Adult, Face to face' or<br>'Specialist Nursing – Continence Services' or<br>'Specialist Nursing – Continence Services, Adult, Face to face' or<br>'Specialist Nursing – Diabetic Nursing/Liaison' or<br>'Specialist Nursing – Diabetic Nursing/Liaison, Adult, Face to face' or<br>'Specialist Nursing – Parkinson's and Alzheimers Nursing/Liaison' or<br>'Specialist Nursing – Stoma Care Services, Adult, Face to face' or<br>'Specialist Nursing – Tissue Viability Nursing/Liaison' or<br>'Specialist Nursing – Tissue Viability Nursing/Liaison, Adult, Face to face' or<br>'Specialist Nursing – Tuberculosis Specialist Nursing'<br>OR<br>Service Reporting Line = '24 hour nursing' or 'Community Nursing' or 'Adult Nursing' or Night Nursing (K&C)' or Twilight/Night Nursing Service' or 'Heart Failure Nursing' or 'Tissue Viability Nursing' or 'TB Nursing' or 'Community Matron' or 'DICE – Community Diabetes Service' or 'Diabetes Service' or 'Continence'
Occupational therapist contacts	ServiceReferenceCostDescription= 'Occupational Therapist, Adult, One to one' OR<br>Service Reporting Line = 'Occupational Therapy'
Physiotherapist contacts	ServiceReferenceCostDescription= 'Physiotherapist' or 'Physiotherapist, Adult, One to one' or 'Physiotherapy services: Adult' or 'Community Rehabilitation Teams' or 'Rehabilitation for Other Disorders' or 'Rehabilitation for other Musculoskeletal Disorders' or 'Rehabilitation for Respiratory Disorders' or 'Rehabilitation for Other Neurological Disorders' OR<br>Service Reporting Line = 'Bedded Rehab – Therapists (H&F, K&C, W)' or 'Brent Rehabilitation Service' or 'Community MSK Physiotherapy service' or 'Community Rehabilitation' or 'Community Rehabilitation (H&F, K&C, W)' or Short Term Rehabilitation' or 'Community IFC MSK Physiotherapy Service' or 'Community Rehab ICE' or 'EDTC – Community Physio' or 'Pulmonary Rehab' or Therapies MS Physio' or 'Community Neuro-Rehabilitation (H&F, K&C, W)' or 'Cardiac Rehabilitation' or 'MSK Physiotherapy' or 'Physio (MSK)' or 'Cardiac Rehab' or 'Community Recovery Service – Neuro Rehab'
Speech and Language therapist contacts	ServiceReferenceCostDescription= 'Speech and Language Therapist, Adult, One to One' or 'SLT – Adult' OR<br>Service Reporting Line = 'Adult SLT'
Rehabilitation teams contacts	OCCUPATIONAL THERAPIST + PHYSIOTHERAPISTS + SPEECH AND LANGUAGE THERAPIST
Primary care practice face to face contacts	Read code v2 = 9N1C., 9N1w., 9NF7., 9NF8., 9k27., 9N1G., 9NFB., 9NFW., 9N1t., 9N1x., 9NF5., 9NF4., 9NF6., 982B., 982C., 9N11., 9N12., 9N1c., 9N1y0, 9N1z., 9c0H., 9N01., 9N0G., 9N7B., 9NV.., 9NY.., 9NY0.,
Primary care practice telephone contacts	Read Code V2 = 9N31., 9b0m., 9b0n., 9b0o, 9N310, 9N310, 9N311, 9N3A, 8CAN., 8CAR0
Primary care practice failed contact	Read code v2 = 9N4.., 9N41%, 9Ni..
Lived in care home	Read code v2=13F6., 13F61, 13FK., 13FX.,

Table 3. Read codes used to derive QoF comorbidities from NHS Digital. Business Rules for Quality and Outcomes Framework (QOF)

Asthma	H33%, H3120, H3B..,173A., NOT (H333., 21262, 212G.)
Atrial Fibrillation	G573% NOT (212R.)
Hypertension	G2..%, G20%, Gyu2., Gyu20, G24..-G2z.., NOT (G24z1, G2400, G2410, G27..)
Diabetes	C10.., C109J, C109K, C10C., C10D., PKyP., C10Q., C10E%, C10F%, C10H%, C10M%, C10N%, C10P% NOT(C10F8)
Congestive heart disease	G3...-G309., G30B.-G330z, G33z.-G3401, G342.-G35X., G38..-G3z.., Gyu3%, NOT(Gyu31, G310.)
COPD	H5832, H4640, H4641, Hyu30, Hyu31, H3..%, H31%, H32%, H36..-H3z.., NOT(H3101, H31y0, H3122, H3y0., H3y1.
Depression	E0013, E0021, E118., E11y2, E11z2, E130, E135., E2003, E291., E2B.., E2B1., Eu204, Eu251, Eu341, Eu412, E112%, E113%, Eu32%, Eu33%, NOT(Eu32a, Eu32B, Eu329, 212S.)
Epilepsy	F1321, SC200, F25%, NOT(F2501, F2504, F2511, F2516, F25y4, F25G., F25H., 21260, 212J, F256%, F258.-F25A.)
Heart Failure	G58%, G1yz1, 662f.-662i.
Peripheral Arterial Disease	G73.., Gyu74, G734., G73y., G73z%, NOT(G73z1)
Rheumatoid Arthritis	N041., N047., N04X., N04y0, N04y2, Nyu11, Nyu12, Nyu1G, Nyu10, G5yA., G5y8., N040%, N042%, NOT(N0420)
Stroke	G65..-G654., G656.-G65zz, G63y0.-G63y1,Gyu62-Gyu66, ZV12D, Fyu55, G6760, G6W.., G6X.., Gyu6F, Gyu6G, G61%, G64%, G66%, NOT(G617., G669.)
Mental Health	E1124, E1134, E11z., E11z0, E11zz, E2122, Eu323, Eu328, Eu333, Eu32A, Eu329, E114.-E117z, E10%, E110%, E111%, E11y%, E12%, E13%, Eu2%, Eu30%, Eu31%, NOT(E11y2, E135.)
Cancer	B0%, B1%, B2%, B3%, B4%, B5%, B6%, Byu%, K1323, K01w1, 68W24, C184., NOT(B677.)
Palliative care	1Z01. , 2JE.. , 2Jf.. , 38VY. , 38Vb. , 38Vd. , 38Ve. , 38Vf. , 38Vg. , 38Vh. , 38Vi. , 8BA2. , 8BAP. , 8BAS. , 8BAT. , 8BAe. , 8BJ1. , 8CM1.% (NOT 8CM15) , 8CM4. , 8CME. , 8CMj. , 8CMk. , 8H6A. , 8H7L. , 8H7g. , 8HH7. , 8IEE. , 9EB5. , 9Ng7. , ZV57C , 8CMQ. , 9NgD. , 9G8.. , 9c0P. , 9c0N. , 8CMW3 , 9K9.. , 9367. , 9c0L0 , 9c0M. , 9NNd. , 8CMb. , 8B2a. , 9NNf0 , 38QH. , 38QK. , 8CMg. , 2Jg.., 9NNq. , 9NNr. , 9NNs.

Table 4. Sensitivity analysis. Multivariate associations between identification of palliative care needs before the last 90 days of life and multiple hospital admissions in the last 90 days of life adjusted by covariables with and without ethnicity

	Original model n=5,804				Model with ethnicity n=4717			
	**RR**	**95%CI**		**p-value**	**RR**	**95%CI**		**p-value**
Identification of palliative care needs before last 90 days								
No	Ref				Ref			
Yes	0.70	0.58	0.85	<0.001	0.76	0.61	0.95	0.014
Age								
<=75	Ref				Ref			
76 to 85	0.98	0.76	1.25	0.853	0.96	0.73	1.27	0.784
76 to 85	0.96	0.75	1.22	0.733	0.92	0.70	1.21	0.557
>=95	0.66	0.47	0.94	0.022	0.63	0.44	0.91	0.014
Gender								
Female	Ref				Ref			
Male	1.55	1.34	1.78	<0.001	1.58	1.36	1.84	<0.001
Lived in care home								
No	Ref				Ref			
Yes	0.57	0.45	0.73	<0.001	0.54	0.40	0.72	<0.001
Number of QoF comorbidities								
0	Ref				Ref			
1	0.69	0.53	0.90	0.007	0.68	0.50	0.92	0.011
2	0.91	0.73	1.12	0.371	0.89	0.71	1.12	0.324
>=3	0.98	0.81	1.18	0.817	0.93	0.77	1.14	0.505
Ethnicity								
White					Ref			
Black					1.15	0.90	1.46	0.252
Asian					1.24	1.02	1.51	0.029
Mixed or other					0.99	0.81	1.21	0.924
